# Interethnic Differences in Bladder Cancer Incidence and the Association between Type 2 Diabetes and Bladder Cancer in the Multiethnic Cohort Study

**DOI:** 10.1158/2767-9764.CRC-22-0288

**Published:** 2023-05-02

**Authors:** David Bogumil, Victoria K. Cortessis, Lynne R. Wilkens, Loïc Le Marchand, Christopher A. Haiman, Gertraud Maskarinec, Veronica Wendy Setiawan

**Affiliations:** 1 Department of Population and Public Health Sciences, Keck School of Medicine of University of Southern California, Los Angeles, California.; 2 Epidemiology Program, University of Hawaii Cancer Center, Honolulu, Hawaii.; 3 Norris Comprehensive Cancer Center, University of Southern California, Los Angeles, California.; 4 Center for Genetic Epidemiology, Keck School of Medicine of University of Southern California, Los Angeles, California.

## Abstract

**Background:**

Research on the association between type 2 diabetes (T2D) and bladder cancer (BCA) risk among non-European ancestry populations is sparse to nonexistent, and most prior studies rely on a single baseline assessment of T2D status.

**Methods:**

We estimated the T2D-BCA association using the Multiethnic Cohort Study of 185,059 men and women in California and Hawaii. Participants were African American, European American, Japanese American, Latin American, and Native Hawaiian, ages 45–75 years at enrollment (1993–1996). T2D was assessed by self-report at baseline, follow-up surveys, and Medicare claims. Cases were identified using Surveillance, Epidemiology and End Results Program cancer registries through 2016. Associations were estimated by race/ethnicity using Cox proportional hazards regression. Adjusted attributable fractions (AAF) and cumulative absolute risk of bladder cancer were estimated across groups.

**Results:**

Over an average 19.7 years of follow-up 1,890 incident bladder cancer cases were diagnosed. Time-varying T2D was associated with bladder cancer in the multiethnic sample (HR = 1.17; 95% confidence interval, 1.05–1.30); however, the HR did not differ by race/ethnicity (*P* = 0.85). The AAF was 4.2% in the multiethnic sample and largest among Native Hawaiians (9.8%). Absolute risk of bladder cancer among European Americans without T2D was higher than all other groups with T2D.

**Conclusion:**

T2D is significantly associated with bladder cancer risk in a multiethnic sample.

**Significance:**

Those with T2D have higher incidence of bladder cancer, regardless of racial/ethnic group. Reducing T2D prevalence could substantially lower bladder cancer incidence among Native Hawaiians due to T2D being more common in this group. High absolute risk of bladder cancer among European Americans, regardless of T2D status, indicates that elevated bladder cancer risk in this group may be due to factors other than T2D. Future studies must explore reasons for this difference in incidence.

## Introduction

In the United States, urinary bladder cancer (BCA) is the eighth most common malignancy among men and the great majority of bladder cancer diagnoses are urothelial carcinoma (1, 2). Age-adjusted incidence is highest in non-Hispanic Whites [23 cases per 100,000 person-years (py)], followed by Blacks (13 per 100,000 py), Hispanic Whites (11 per 100,000 py), and Asian Pacific Islanders (9.5 per 100,000 py; 2, 3). Age, being male, a history of smoking, certain occupational exposures, and obesity are common risk factors for bladder cancer (4), with type 2 diabetes (T2D) being one of the most common risk factors (5).

Epidemiologic studies on the association between T2D and bladder cancer risk in non-European ancestry populations are sparse to nonexistent depending on the racial/ethnic group of interest, with two prospective cohorts reporting associations in admixed samples in the United States (6, 7). T2D is a common risk factor for bladder cancer, associated with a 20%–30% increase in risk (5, 8–10), and both the prevalence of T2D and the incidence of bladder cancer differ dramatically between racial/ethnic groups (3, 11, 12).

T2D prevalence is estimated to be 14% nationally; however, estimates vary based on method of measurement and race/ethnicity (12). Mexican Americans, a subset of Hispanics, have the highest prevalence of T2D at 22% in the United States, followed by non-Hispanic Asians (19%), non-Hispanic Blacks (18%), and non-Hispanic Whites (14%; 12). Given the high prevalence of T2D among non-Whites, we would expect T2D to account for a greater number of bladder cancer cases in these racial/ethnic groups, unless the association is considerably stronger among non-Hispanic Whites. However, relative risks, attributable fractions, and absolute risks of bladder cancer associated with T2D have not been reported across racial/ethnic populations; the impact of T2D on bladder cancer risk within these groups was unknown at the outset of this work.

Beyond this gap in the literature, most U.S. cohort studies reporting on the T2D-BCA association have determined T2D status from a single baseline measure (5). The National Health and Nutrition Examination Survey estimated an additional 3.4% in T2D prevalence, 6.2% among Mexican Americans, when using elevated HbA1c and fasting plasma glucose levels beyond self-report (12), showing that multiple measures of T2D may increase the sensitivity of exposure assessment.

To provide information on the association of T2D and bladder cancer risk among non-White populations, we used the Multiethnic Cohort Study (MEC) to assess this relationship in African Americans, European Americans, Japanese Americans, Latin Americans, and Native Hawaiians. Because of the change in T2D status during follow-up and underreporting of T2D status, we assessed associations using two different measures of T2D. For the first measure, we used diabetes status taken from the baseline self-report survey responses. For the second, we used a variable derived from self-report at baseline, follow-up self-report, and Medicare claims data. We assessed heterogeneity of associations by race/ethnicity, examined how eliminating T2D could affect bladder cancer risk using attributable fractions, and estimated absolute risk of bladder cancer within populations by T2D status.

## Materials and Methods

### Study Participants

We used MEC data to estimate the association between T2D status and incident urothelial carcinoma of the bladder. The MEC is a population-based prospective cohort study of over 215,000 men and women between the age of 45 and 75 years residing in Southern California and Hawaii at baseline. Participants were enrolled between 1993 and 1996. Details of recruitment and data collection have been described previously (13). Participants from five major racial/ethnic groups (African American, European American, Japanese American, Latin American, and Native Hawaiian) were identified using the Department of Motor Vehicles, Health Care Financing Administration files, and voter registration lists. Participants were mailed a letter explaining the study goals and a 14-page questionnaire which served to collect self-reported diet, demographic characteristics, smoking history, anthropometric measures, reproductive factors among females, chronic medical conditions, and physical activity. The University of Hawaii (Honolulu, Hawaii) and University of Southern California (Los Angeles, CA) Institutional Review of Boards (IRB) considered the cover letter and the return of the questionnaire as proof of written informed consent. The reviewal and approval from IRBs is in accordance with the Department of Health and Human Services regulations in 45 CFR 46. 45 CFR 46 includes subpart A, the Common Rule, which provides protections for research subjects. In addition, subparts B, C, and D provide protections for specific populations involved in research and subpart E provides requirements for IRB registration.

### Assessment of T2D Status

We report the association between T2D and bladder cancer risk, for two different T2D definitions. The first T2D variable is self-reported T2D status on the baseline questionnaire (Q1), referred to as *Baseline T2D*. The question asked, “Has your doctor ever told you that you had any of the following”, with “diabetes (high blood sugar)” as an option. The second T2D variable, referred to as *Any T2D*, is a composite variable that defines T2D using the baseline T2D status, self-report on follow-up questionnaires sent every 5 years (Q2–Q5), self-report of T2D medication usage among MEC biorepository participants, and Medicare claims data (1990–2016) for fee-for-service (FFS) participants. Only FFS data were used as managed care plans do not offer enough detail for T2D classification (14). FFS T2D status was defined based on the following Chronic Condition Warehouse criteria: (i) at least one inpatient, skilled nursing facility, or home health claim; or (ii) two hospital outpatient or carrier claims for T2D during a 2-year period (15). The date of onset for the *Any T2D* exposure was based on the first report of T2D using any of the available data sources.

### Bladder Cancer Assessment

Incident bladder cancer cases were identified through cohort linkage to the California Cancer Registry and Hawai`i Tumor Registry, statewide registries that are members of the NCI's Surveillance, Epidemiology and End Results Program (SEER). Bladder cancer was defined as urothelial carcinoma, also termed transitional cell carcinoma, of the bladder. These cases were identified using ICD-O-3 site codes, C67.0–C67.9, excluding histology codes 9050–9055, 9140, and 9590–9992; *in situ* and invasive tumors are included. Additional linkage to the National Death Index and state death certificate files were used to obtain information on vital status and cause of death. Participants with missing T2D status at baseline, smoking status, alcohol consumption, body mass index (BMI), and bladder cancer prior to cohort entry were removed from analysis. There were 185,059 MEC participants and 1,890 incident bladder cancer cases over the study period (Supplementary Fig. S1).

### Statistical Analysis

We used Cox proportional hazards regression of bladder cancer to estimate the association with both T2D definitions. Age, measured in days, was used as the timescale for analysis as it has been shown to produce the least biased measures of association in cohort study designs and adjusts for age (16, 17). Because T2D (*Any T2D*) exposure status can change over the study period, this variable was modeled as a time-varying exposure, where the participant was classified as exposed at first report of T2D. Participants were censored at time of bladder cancer diagnosis, death, or end of follow-up (December 31, 2016). The Efron approximation was used in the case of tie events. We present HRs and Wald 95% confidence intervals (CI) for all estimates. Statistical significance was determined by no overlap of the 95% CI with the null hazard ratio (HR) value of one. The proportional hazards assumption was assessed by testing for a constant hazard rate for each variable over age. A global test for all levels of each variable was used to determine whether the variable violated the assumption. All exposure variables and confounders were tested (Supplementary Table S1).

We adjusted for confounders using baseline questionnaire data and conducted a sensitivity analysis using time-varying smoking to ensure results did not change. We included the following categorical confounders: pack-years smoked (0, 1–10, 11–20, 21–30, 31–40, >40), number of cigarettes smoked per day among ever smokers (5 cigarettes or less, 6–10 cigarettes, 11–20, 21–30, 31, or more), alcohol consumption over the prior year (<1 drink/month, ≤1 drink/day, >1 drinks/day), sex (male, female), race/ethnicity (European American, African American, Japanese American, Latin American, Native Hawaiian), estrogen use among females (ever, missing, never), progesterone use among females (ever, missing, never), parity among females (nulliparous, 1, 2–3, ≥4), menopause status among females (premenopausal, natural menopause, surgical menopause, other surgery causing periods to stop, period stopped for unknown reason, missing). BMI (kg/m^3^) and smoking status (ever, never, current) were included as strata variables due to their proportional hazards violations. Categorical birth year was also included as a strata variable to adjust for possible cohort effects.

We produced separate results by race/ethnicity and covariates associated with the exposure or outcome to assess heterogeneity of associations by these variables. We used likelihood ratio tests to determine statistically significant differences within each variable. Null models included the variable of interest as a strata term and full models included the variable of interest as a strata and interaction term with the T2D variable. Because it may take years for T2D to affect risk of bladder cancer, we conducted sensitivity analyses censoring bladder cancer cases that occurred within 2 years of incident T2D (or 2 years from baseline) as noncases. We also present results in a subcohort restricted to MEC participants with Medicare FFS enrollment.

To determine the effect of T2D on bladder cancer across racial/ethnic groups, we estimated adjusted attributable fractions (AAF) for each group, using the R package *AF* (18). The AAF statistic represents the percentage of bladder cancer cases that would be eliminated in the MEC before age 85 if the exposure *Any T2D* were eliminated, after adjusting for confounding variables (18, 19).

To illustrate how T2D affects absolute risk of bladder cancer across race/ethnicity, we estimated incidence of bladder cancer per 100,000 participants within race/ethnicity and *Any T2D* status. We used a log-linear Poisson model with case counts as a function of age and time-varying T2D status. We fit models separately for each ethnic/racial group, using py within each group as an offset term (Supplementary Table S2). To generate the summary data needed to fit the Poisson model, we tabulated individual level data by race/ethnicity, age (5-year groups), and time-varying T2D status. Within tabulated strata, we calculated time-dependent log-transformed age, py, and number of cases. The fit risk models were then used to predict age-specific incidence and cumulative risk percent by race/ethnicity and exposure status, conditional on survival and not developing bladder cancer.

### Data Availability

The data generated in this study are available within the article and its Supplementary Data but may be obtained through a MEC data request application at (https://www.uhcancercenter.org/for-researchers/mec-data-sharing).

## Results

### Sample Characteristics

The 185,059 participants were followed over 19.7 years on average, accumulating 3,449,103 py of follow-up time. There were 1,890 cases of incident bladder cancer (256 African American, 665 European American, 547 Japanese American, 311 Latin American, 111 Native Hawaiian). The age-adjusted incidence rate of bladder cancer, left truncated at age 45, was 35.4 per 100,000 py. European Americans experienced the highest rate (52.3 per 100,000 py), followed by Native Hawaiians and Japanese Americans (34.5 per 100,000 py, 33.6 per 100,000 py), African Americans (30.5 per 100,000 py), and Latin Americans (24.2 per 100,000 py; Supplementary Table S3).

In the multiethnic sample, 11.7% of the cohort reported having T2D at baseline (Table 1), in comparison with 35.3% being classified as having T2D at baseline or during the follow-up period. Data availability across all sources was greatest among Japanese Americans (28.5%), followed by Native Hawaiians (23.9%), European Americans (18.6%), Latin Americans (18.1%), and African Americans (14.7%; Supplementary Fig. S2). A similar pattern across racial/ethnic groups is seen for follow-up survey response rates (Supplementary Fig. S3). Across both T2D definitions the pattern of lowest prevalence among European American participants is consistent (*Baseline T2D*: 5.9%, *Any T2D*: 24.3%), followed by Japanese Americans (*Baseline T2D* 10.5%, *Any T2D*: 35.7%), with the remaining racial/ethnic groups having similar prevalence for both measures at around 15% and 40% for *Baseline T2D* and *Any T2D*, respectively.

**TABLE 1:** Cohort characteristics

	Multiethnic (*n* = 185,059)		African American (*n* = 30,481)		European American (*n* = 45,995)		Japanese American (*n* = 53,216)		Latin American (*n* = 41,995)		Native Hawaiian (*n* = 13,372)	
	*n*	%	*n*	%	*n*	%	*n*	%	*n*	%	*n*	%
Bladder cancer cases	1,890	1.0	256	0.8	665	1.4	547	1.0	311	0.7	111	0.8
Sex												
Male	83,766	45.3	11,129	36.5	21,212	46.1	25,187	47.3	20,375	48.5	5,863	43.8
Female	101,293	54.7	19,352	63.5	24,783	53.9	28,029	52.7	21,620	51.5	7,509	56.2
Baseline T2D												
No	163,453	88.3	25,699	84.3	43,280	94.1	47,617	89.5	35,463	84.4	11,394	85.2
Yes	21,606	11.7	4,782	15.7	2,715	5.9	5,599	10.5	6,532	15.6	1,978	14.8
Any T2D												
No	119,781	64.7	18,466	60.6	34,822	75.7	34,212	64.3	24,422	58.2	7,859	58.8
Yes	65,278	35.3	12,015	39.4	11,173	24.3	19,004	35.7	17,573	41.8	5,513	41.2
BMI (kg/m^2^)												
≤18.5	3,215	1.7	305	1	785	1.7	1,787	3.4	225	0.5	113	0.8
18.6–24.9	73,850	39.9	7,913	26	20,405	44.4	30,302	56.9	11,783	28.1	3,447	25.8
25–29.9	71,188	38.5	12,492	41	16,722	36.3	17,391	32.7	19,556	46.6	5,027	37.6
≥30	36,806	19.9	9,771	32	8,083	17.6	3,736	7	10,431	24.8	4,785	35.8
Smoking status												
Never	83,294	45	11,823	38.8	18,110	39.4	26,925	50.6	21,149	50.4	5,287	39.5
Past	72,100	39	11,790	38.7	20,317	44.2	20,040	37.7	14,887	35.4	5,066	37.9
Current	29,665	16	6,868	22.5	7,568	16.4	6,251	11.7	5,959	14.2	3,019	22.6
Pack-years												
0	36,348	19.6	6,870	22.5	8,353	18.1	7,796	14.6	10,967	26.1	2,362	17.7
(0–10]	33,113	17.9	6,973	22.9	8,020	17.4	9,398	17.7	5,892	14	2,830	21.2
(10–20]	8,706	4.7	1,595	5.2	2,605	5.7	2,447	4.6	1,263	3	796	5.9
(20–30]	12,384	6.7	1,974	6.5	4,128	9	3,567	6.7	1,603	3.8	1,112	8.3
(30–40]	83,294	45	11,823	38.8	18,110	39.4	26,925	50.6	21,149	50.4	5,287	39.5
40+	11,214	6.1	1,246	4.1	4,779	10.4	3,083	5.8	1,121	2.7	985	7.4
Alcohol consumption												
<1 drink/month	98,836	53.4	17,838	58.5	17,226	37.5	34,175	64.2	22,222	52.9	7,375	55.1
≤1 drink/day	53,227	28.8	8,519	28	15,747	34.2	11,661	21.9	13,678	32.6	3,622	27.1
>1 drinks/day	32,996	17.8	4,124	13.5	13,022	28.3	7,380	13.9	6,095	14.5	2,375	17.8
Birthyear												
1918–1922	24,109	13	5,079	16.7	5,904	12.8	8,413	15.8	3,806	9.1	907	6.8
1923–1927	32,440	17.5	6,876	22.5	7,027	15.3	10,715	20.1	6,415	15.3	1,407	10.5
1928–1932	32,450	17.5	4,897	16.1	7,025	15.3	9,603	18.1	8,991	21.4	1,934	14.5
1933–1937	28,761	15.6	4,518	14.8	6,592	14.3	7,145	13.4	8,459	20.1	2,047	15.3
1938–1942	29,770	16.1	4,331	14.2	7,918	17.2	6,585	12.4	8,506	20.3	2,430	18.2
1943–1953	37,529	20.3	4,780	15.7	11,529	25.1	10,755	20.2	5,818	13.8	4,647	34.7

NOTE: Baseline sample characteristics, stratified by race/ethnicity.

Abbreviation: T2D, type 2 diabetes.

Most bladder cancer cases were classified as SEER stage 0 (*in situ*, noninvasive, intraepithelial), among Native Hawaiians (49%), Japanese American (47%), European American (45%), Latin American (31%), and African American (24%). Stage 1 (localized only) was more common than stage 0 among African Americans (57%) and Latin Americans (48%), in contrast to the other racial/ethnic groups (Supplementary Fig. S4).

### Type 2 Diabetes—Bladder Cancer Association

**FIGURE 1:**
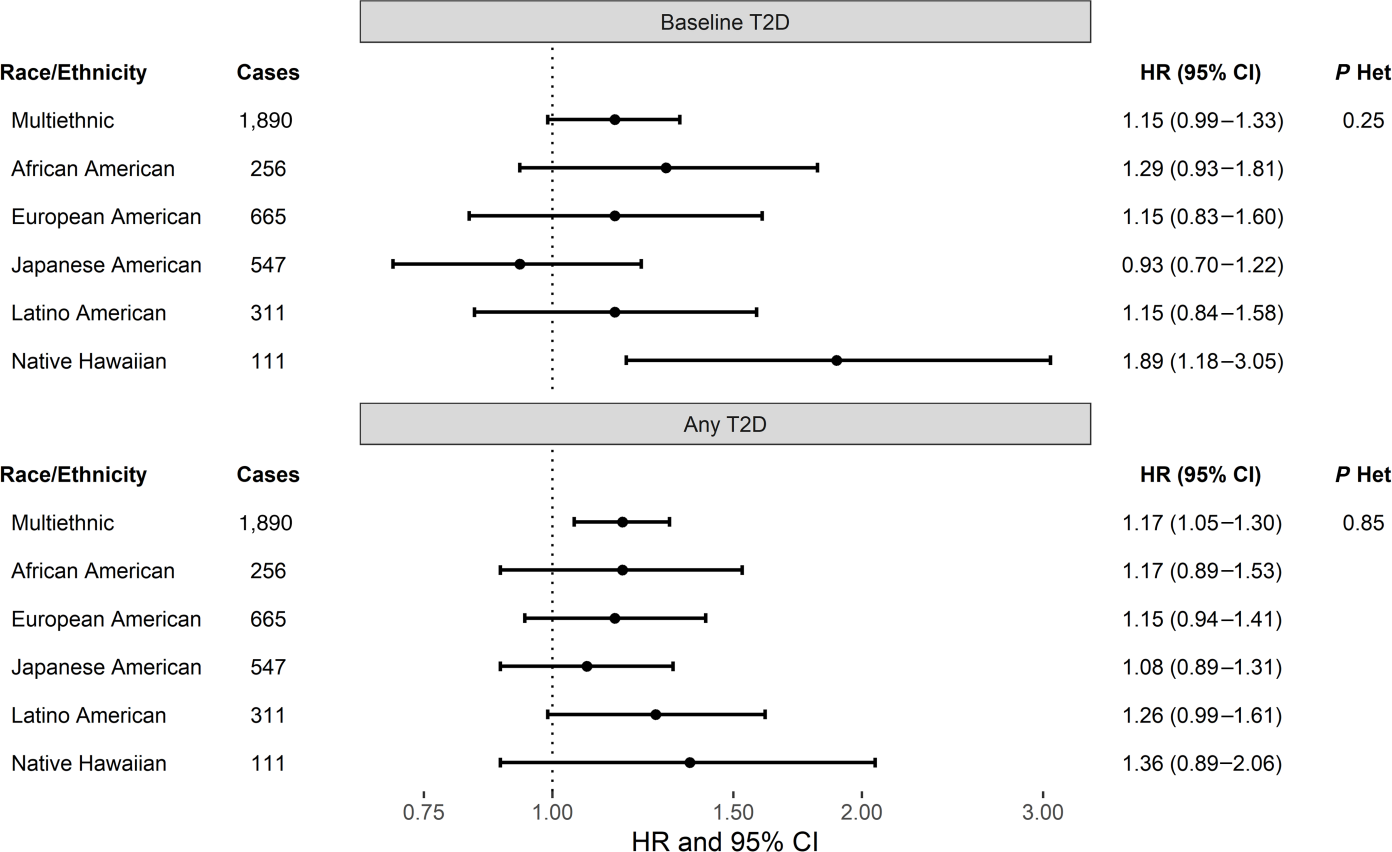
Association between T2D and bladder cancer in the multiethnic sample and by race/ethnicity, stratified by T2D assessment method. Models adjusted for pack-years smoked (0, 1–10, 11–20, 21–30, 31–40, >40), alcohol consumption (<1 drink/month, ≤1 drink/day, >1 drinks/day), sex (male, female), number of cigarettes smoked per day among smokers (5 cigarettes or less, 6–10 cigarettes, 11–20, 21–30, 31, or more), race/ethnicity (European American, African American, Japanese American, Latin American, Native Hawaiian), and reproductive factors among females. BMI (kg/m^3^) and smoking status (ever, never, current) were included as strata variables due to their proportional hazard violation. Categorical birthyear was also included as a strata variable to adjust for possible cohort effects. CI, confidence interval; HR, hazard ratio; *P* het: *P*-heterogeneity for likelihood ratio test for difference in association across race/ethnicity groups.

Using Baseline T2D status, there was 1.15 (95% CI, 0.99–1.33) times the rate of bladder cancer among those with, compared with those without, T2D in the multiethnic sample (Fig. 1), with little indication of heterogeneity of association across racial/ethnic groups (*P* Het = 0.25). Within groups, the association was statistically significant only among Native Hawaiians (HR = 1.89; 95% CI, 1.18–3.05). *Any T2D* was also significantly associated with a similar magnitude in the multiethnic sample (HR = 1.17, 95% CI, 1.05–1.30), with little indication of heterogeneity by race/ethnicity (P Het = 0.85).

**FIGURE 2:**
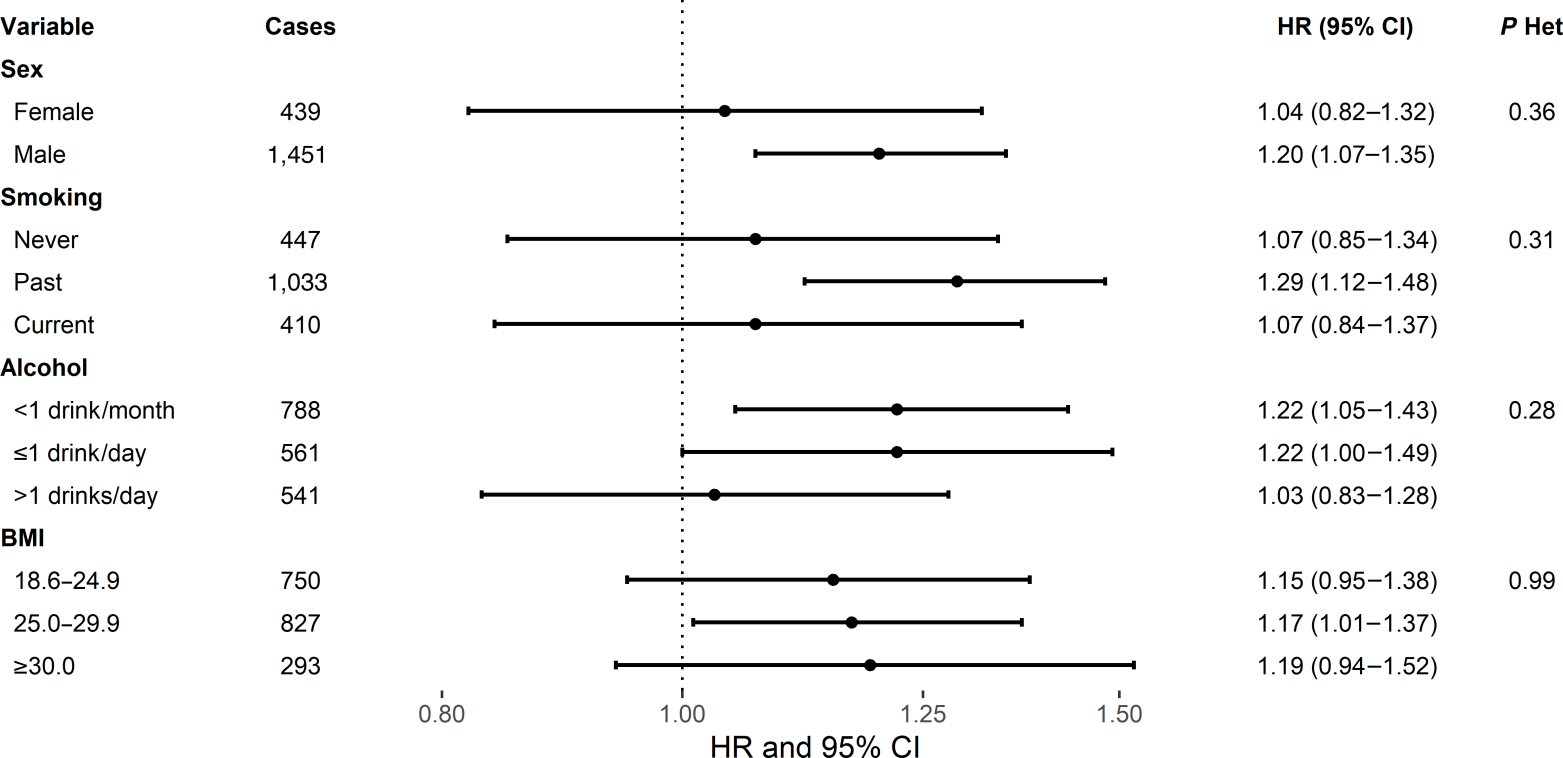
Association between *Any T2D* and bladder cancer in the multiethnic sample stratified by sex, smoking status, alcohol consumption, and BMI. Models adjusted for pack-years smoked (0, 1–10, 11–20, 21–30, 31–40, >40), alcohol consumption (<1 drink/month, ≤1 drink/day, >1 drinks/day), sex (male, female), number of cigarettes smoked per day among smokers (5 cigarettes or less, 6–10 cigarettes, 11–20, 21–30, 31, or more), race/ethnicity (European American, African American, Japanese American, Latin American, Native Hawaiian), and reproductive factors among females. BMI (kg/m^3^) and smoking status (ever, never, current) were included as strata variables due to their proportional hazard violation. Categorical birthyear was also included as a strata variable to adjust for possible cohort effects. CI, confidence interval; HR, hazard ratio; *P* het: *P*-heterogeneity for likelihood ratio test for difference in association across covariate levels.

In the covariate-stratified analysis, we observed no significant heterogeneity of the *Any T2D*–BCA association by sex, smoking status, alcohol consumption, or BMI (Fig. 2). Within covariate strata, the association was strongest among males (HR = 1.20; 95% CI, 1.07–1.35) and past smokers (HR = 1.29; 95% CI, 1.12–1.48). Because of the high prevalence of past smoking among cohort members (39%), and smoking being a major risk factor for bladder cancer, the majority of bladder cancer diagnoses were among past smokers in each racial/ethnic group (African American: 57%, European American: 56%, Japanese American: 53%, Latin American: 51%, Native Hawaiian: 55%).

In sensitivity analyses that censored bladder cancer cases diagnosed within 2 years of incident T2D (Supplementary Figs. S5 and S6), the risk estimates in the multiethnic sample (*Any T2D* HR = 1.18; 95% CI, 1.05–1.32) and within racial/ethnic groups changed very little. We also found similar estimates among the subcohort with Medicare FFS enrollment history (*Any T2D* HR = 1.16; 95% CI, 1.01–1.33; Supplementary Fig. S7). In addition to these sensitivity analyses, we present results of analyses stratified by sex and stage, and analyses restricted to never smokers (Supplementary Figs. S8–S11). Associations were weaker among females in European Americans (HR = 0.96), Japanese Americans (HR = 0.99), and Native Hawaiians (HR = 0.66; Supplementary Fig. S8) and in analyses stratified on both sex and each of smoking status, alcohol consumption, and BMI (Supplementary Fig. S9). Associations were also weaker among never smokers in most racial and ethnic groups, except for African Americans and Japanese Americans, where associations of bladder cancer with *Any T2D* were slightly stronger in never smokers than among all participants (Supplementary Fig. S11).

### AAFs

The estimate of the bladder cancer AAF for *Any T2D* in the multiethnic sample was 4.2% (95% CI, 1.2–7.3). Within racial/ethnic groups, the AAF was highest among Native Hawaiians (9.8%; 95% CI, −3.9 to 23.4) followed by Latin Americans (7.1%; 95% CI, −1.0 to 15.1), African Americans (5.3%; 95% CI, −3.5 to 14.0), European Americans (3.0%; 95% CI, −1.4 to 7.4), and Japanese Americans (2.9%; 95% CI, −3.0 to 8.7) with considerable overlap of CIs.

### Absolute Risk

**FIGURE 3:**
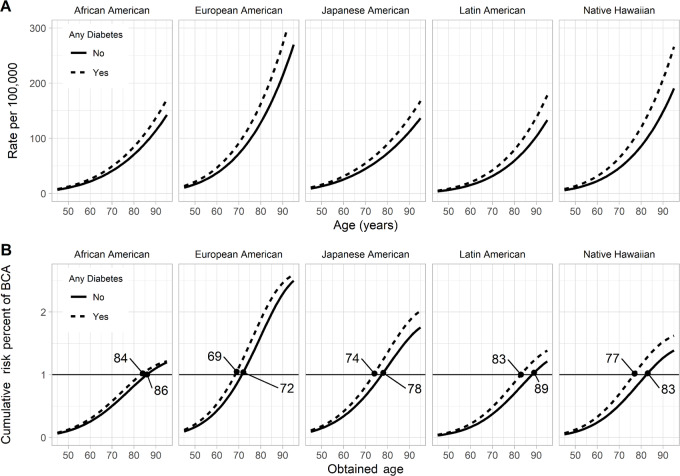
**A,** Incidence of bladder cancer by *Any T2D* exposure status, per 100,000 py. Each line represents age-specific incidence, with the solid line indicating incidence among those without *Any T2D* and the dotted line indicating incidence among those with *Any T2D*. Incidence was estimated using Poisson regression model estimates within groups (Supplementary Table S2). Data were tabulated and fit using race/ethnicity, exposure status, and 5-year age categories. **B,** Cumulative risk percent of bladder cancer based on age obtained by race/ethnicity and *Any T2D* status, with the solid line indicating incidence among those without *Any T2D* and the dotted line indicating incidence among those with *Any T2D*. Age at which 1% lifetime risk is marked by the horizontal line and indicated age.

We observed considerable differences in incidence between racial/ethnic groups, and these mirror the age-adjusted incidence estimates (Supplementary Table S3). Model-based bladder cancer incidence estimates for those with and without T2D are shown in Fig. 3A. Bladder cancer incidence among European Americans is notably higher than all other racial/ethnic groups. Even among European American participants without T2D, bladder cancer incidence is higher than those with T2D in all other racial/ethnic groups. Similarly, on the cumulative risk scale (Fig. 3B), European American participants with T2D achieved 1% risk of bladder cancer at the earliest age, 69 years old, followed by Japanese Americans (74 years), Native Hawaiians (77 years), Latin Americans (83 years), and African Americans (84 years).

## Discussion

In this prospective analysis, time-varying T2D status (*Any T2D*) was associated with a significant 17% increase in risk of bladder cancer. Recently published meta-analyses on this association reported a 23%–29% increase in risk of incident bladder cancer and bladder cancer mortality (5, 10). The latest study restricting to cohort studies identified a 23% increase in risk (relative risk [RR] = 1.23; 95% CI, 1.12–1.35) of bladder cancer diagnosis or death among participants with T2D (5). Included in this previous analysis was a MEC study that followed participants from 1993 to 2004 and reported an HR estimate of 1.25 (95% CI, 1.04–1.50) between self-reported T2D at baseline and incident urothelial cancer, which included cancers outside the bladder and nonurothelial carcinomas (7). In contrast to this previous analysis, we found weaker associations for European Americans (HR = 1.15 vs. 1.38) and African Americans (HR = 1.17 vs. 1.40). We detected a much stronger association among Latin Americans (HR = 1.26 vs. 1.03). We were also able to estimate an association among Native Hawaiians (HR = 1.36), estimate AAFs, and absolute risk. Similar to this analysis of urothelial cancer, we observed no heterogeneity of associations by race/ethnicity for both T2D definitions.

Our covariate analysis identified no significant modification of the association of *Any T2D* with bladder cancer by sex, smoking status, alcohol consumption, or BMI. Within strata defined by sex, the association was considerably stronger and achieved statistical significance only among males (male HR = 1.20, female HR = 1.04). Sex-specific associations estimated in a meta-analysis identified similar values for each sex (male RR = 1.23, female RR = 1.24). Authors considered control of smoking to be important in estimation among males, who are at higher risk for bladder cancer and have a higher prevalence of smoking, a major risk factor for bladder cancer (5). As a sensitivity analysis using restriction to remove confounding by smoking, we estimated results among never smokers. In this analysis, we find a weaker association (HR = 1.07; 95% CI, 0.85–1.34). Assuming that smoking does not modify associations of T2D with bladder cancer, it is possible that residual confounding may have biased our results upward from the null.

Possible mechanisms for the T2D-BCA association have been previously reviewed in detail (5, 20–22) and include the following: (i) impaired immune system function from hyperglycemia (5, 20), (ii) inflammation resulting in oxidative damage causing DNA mutations (20, 22), (iii) increased insulin-like growth factor among participants using insulin that may stimulate cell proliferation and inhibit cell apoptosis (5, 21, 22), (iv) increased risk of urinary tract infections from urinary glucose causing bladder cancer (5), (v) medications that increase activation of insulin receptors or increase insulin sensitivity (i.e., sulfonylureas and thiazolidinediones; 20–22). Although we did not detect a statistically significant difference in the association by race/ethnicity, differences in estimates across groups could be attributed to differences in the prevalence of these mechanistic components, T2D severity, and level of glucose control across groups. For example, the severity and cumulative exposure of T2D is greater among Native Hawaiians, characterized by an average 5 years earlier onset and 6.7 times the rate of mortality when T2D is considered the underlying cause of death (23). This longer and more intense exposure could explain the stronger association we observed among Native Hawaiians.

To better understand the public health impact of T2D on bladder cancer within these populations, we estimated AAFs and absolute risk among those with and without T2D. In the multiethnic sample, we estimated an AAF to be 4.2%, meaning 4.2% of the bladder cancer cases could be prevented by age 85 if T2D were eliminated at baseline. Among Native Hawaiians, the high prevalence of *Any T2D* (41.2%) and high HR (1.36) resulted in the greatest percent of bladder cancer cases that could have been prevented (9.8%) by T2D prevention.

Mirroring the age-adjusted incidence rates in our analysis and rates observed in SEER (3), European Americans had the highest age-specific incidence rates among all groups. On the cumulative risk scale, European Americans had the youngest age to achieve 1% risk of bladder cancer (69 years old) among those with T2D. Native Hawaiians, who had the strongest T2D-BCA association, reached this same cumulative risk 8 years later. We estimated that by 58 years of age, the lifetime risk of bladder cancer among European Americans without T2D surpasses that of any other racial/ethnic group with T2D. Given these results, T2D is unlikely to be the risk factor leading to increased risk of bladder cancer among European Americans.

By comparing the T2D-BCA association according to two separate definitions, we found the time-varying T2D HR (*Any T2D*) to have greater precision, meaning narrower CIs, and a larger magnitude. The *Any T2D*–BCA association may more accurately represent the true association. Using multiple T2D measurements will increase the sensitivity of the measure, identifying more participants with T2D. If this is a more valid measure, the *Baseline T2D* association may be weaker due to misclassification of participants with T2D being classified as not having T2D. In contrast, the high prevalence of diabetes we observed at the end of the study period when using the *Any T2D* definition may represent cases of prediabetes or metabolic syndrome from Medicare claims of procedures used to rule out diabetes. Both prediabetes and metabolic syndrome are associated with “all cancer” and bladder cancer, respectively (10, 24). Unintentional inclusion of these phenotypes in the *Any T2D* group may alter the magnitude and statistical power of the association in comparison with the *Baseline T2D* definition.

### Strengths

There are several strengths to this study. We used a large, multiethnic, prospective study and adjusted for a comprehensive list of confounders. We estimated associations for five major racial/ethnic groups and estimated race/ethnic-specific associations for many understudied groups, including Native Hawaiians who have a high prevalence of T2D and have not been studied in this context. We presented associations for both self-reported T2D status and a more detailed follow-up measure based on self-report, medication usage, and Medicare claims data. Using absolute risk models, we presented estimated incidence rates of bladder cancer by race/ethnicity and T2D status, results which have not been presented before. We also conducted several sensitivity analyses to ensure the robustness of our associations.

### Limitations

There are a few limitations to this study. First, we do not differentiate between type 1 and type 2 diabetes in the analysis; however, due to the low prevalence of type 1 diabetes in this age group this likely does not appreciably affect results. Second, *Any T2D* was defined using Medicare claims data, which was only available for FFS participants who were 65 years or older. *Any T2D* was also based on self-report during follow-up surveys, where response rates decreased in each successive survey. Third, younger participants, participants with more follow-up, and those who decided to complete later questionnaires have more opportunities to be classified as having T2D. Although this limitation exists, it did not appear to change impression of these results from what we observed using baseline self-report only. Finally, without preenrollment information on T2D onset, participants reporting T2D at baseline may differ in cumulative exposure time.

## Conclusion

We present a comprehensive analysis on the association between T2D and bladder cancer across multiple racial and ethnic groups using both baseline assessed T2D and time-varying T2D that incorporates additional assessments based on follow-up questionnaires and Medicare claims data. The analysis detected an association between time-varying T2D and bladder cancer. We observed the greatest absolute risk among European Americans with and without T2D. Future research is needed to replicate the association among Native Hawaiians or other Pacific Islanders, for whom this first report shows a high portion of cases to be attributable to T2D. Finally, as T2D does not explain the increased risk of bladder cancer among European Americans, future research needs to explore variables responsible for this disparity, which could inform primary prevention strategies for all racial/ethnic groups.

## Supplementary Material

Supplementary Figure S1 Supplementary Figure 1: MEC participant exclusion, showing removal of participants based on data missingness.

Supplementary Figure S2 Supplementary Figure 2: Surveillance, Epidemiology, and End Results Program stage distribution of bladder cancer cases by race/ethnicity. 0: In situ: noninvasive, intraepithelial; 1: Localized only; 2: Regional by direct extension only; 3: Regional lymph node(s) involved only; 4: Regional by BOTH direct extension AND regional lymph node(s) involved; 5: Regional, NOS; 7: Distant site(s)/lymph node(s) involved; 8: Unknown if extension or metastasis; M: Missing.

Supplementary Figure S3 Supplemental Figure 3: Distribution of MEC participation across participation opportunities by race/ethnicity, represented as percentage by race/ethnicity. BL: baseline questionnaire; FU QX: follow-up questionnaire; BR: biorepository questionnaire; CMS: Medicare FFS.

Supplementary Figure S4 Supplemental Figure 4: Response rates to follow-up questionnaire among MEC participants who returned the baseline questionnaire and were alive for each respective survey by race/ethnicity. As an example, on the 3rd questionnaire, 44.3% of living African American participants returned a questionnaire. Bar heights shorten based on number of participants alive during the survey collection period. QX: Questionnaire.

Supplementary Figure S5 Supplementary Figure 5: Race/ethnicity specific associations between Any T2D and BCA, with censorship of BCA cases that occurred within 2 years of incident T2D as non-cases. Models adjusted for pack-years smoked (0, 1-10, 11-20, 21-30, 31-40, >40), alcohol consumption (<1 drinks/month, ≤1 drinks/day, >1 drinks/day), sex (male, female), number of cigarettes smoked per day among smokers (5 cigarettes or less, 6-10 cigarettes, 11-20, 21-30, 31 or more), race/ethnicity (European American, African American, Japanese American, Latin American, Native Hawaiian), and reproductive factors among females. Body mass index (BMI, kg/m3) and smoking status (ever, never, current) were included as strata variables due to their proportional hazard violation. Categorical birthyear was also included as a strata variable to adjust for possible cohort effects. CI: Confidence Interval; HR: Hazard Ratio; P het: P- heterogeneity for likelihood ratio test for difference in association across race/ethnicity groups.

Supplementary Figure S6 Supplementary Figure 6 Association between Any T2D and bladder cancer in the multiethnic sample stratified by sex, smoking status, alcohol consumption, and BMI, with censorship of BCA cases that occurred within 2 years of incident T2D as non-cases. Models adjusted for pack-years smoked (0, 1-10, 11-20, 21-30, 31-40, >40), alcohol consumption (<1 drinks/month, ≤1 drinks/day, >1 drinks/day), sex (male, female), number of cigarettes smoked per day among smokers (5 cigarettes or less, 6-10 cigarettes, 11-20, 21-30, 31 or more), race/ethnicity (European American, African American, Japanese American, Latin American, Native Hawaiian), and reproductive factors among females. Body mass index (BMI, kg/m3) and smoking status (ever, never, current) were included as strata variables due to their proportional hazard violation. Categorical birth year was also included as a strata variable to adjust for possible cohort effects. CI: Confidence Interval; HR: Hazard Ratio; P het: P- heterogeneity for likelihood ratio test for difference in association across covariate levels.

Supplementary Figure S7 Supplementary Figure 7: Association between T2D and BCA in the multiethnic sample and by race/ethnicity, stratified by T2D assessment method, restricted to MEC participants with Medicare FFS enrollment. Models adjusted for pack-years smoked (0, 1-10, 11-20, 21-30, 31-40, >40), alcohol consumption (<1 drinks/month, ≤1 drinks/day, >1 drinks/day), sex (male, female), number of cigarettes smoked per day among smokers (5 cigarettes or less, 6-10 cigarettes, 11-20, 21-30, 31 or more), race/ethnicity (European American, African American, Japanese American, Latin American, Native Hawaiian), and reproductive factors among females. Body mass index (BMI, kg/m3) and smoking status (ever, never, current) were included as strata variables due to their proportional hazard violation. Categorical birthyear was also included as a strata variable to adjust for possible cohort effects. CI: Confidence Interval; HR: Hazard Ratio; P het: P- heterogeneity for likelihood ratio test for difference in association across race/ethnicity groups.

Supplementary Figure S8 Supplementary Figure 8: Association between Any T2D and BCA stratified by sex and race/ethnicity. Models adjusted for pack-years smoked (0, 1-10, 11-20, 21-30, 31-40, >40), alcohol consumption (<1 drinks/month, ≤1 drinks/day, >1 drinks/day), sex (male, female), number of cigarettes smoked per day among smokers (5 cigarettes or less, 6-10 cigarettes, 11-20, 21-30, 31 or more), race/ethnicity (European American, African American, Japanese American, Latin American, Native Hawaiian), and reproductive factors among females. Body mass index (BMI, kg/m3) and smoking status (ever, never, current) were included as strata variables due to their proportional hazard violation. Categorical birth year was also included as a strata variable to adjust for possible cohort effects. CI: Confidence Interval; HR: Hazard Ratio; P het: P- heterogeneity for likelihood ratio test for difference in association by sex.

Supplementary Figure S9 Supplementary Figure 9: Sex stratified analysis for the association between Any T2D and BCA. Models adjusted for pack-years smoked (0, 1-10, 11-20, 21-30, 31-40, >40), alcohol consumption (<1 drinks/month, ≤1 drinks/day, >1 drinks/day), number of cigarettes smoked per day among smokers (5 cigarettes or less, 6-10 cigarettes, 11-20, 21-30, 31 or more), race/ethnicity (European American, African American, Japanese American, Latin American, Native Hawaiian), and reproductive factors among females. Body mass index (BMI, kg/m3) and smoking status (ever, never, current) were included as strata variables due to their proportional hazard violation. Categorical birthyear was also included as a strata variable to adjust for possible cohort effects. CI: Confidence Interval; HR: Hazard Ratio; P het: P- heterogeneity for likelihood ratio test for difference in association across covariate levels.

Supplementary Figure S10 Supplementary Figure 10: Association between Any T2D and BCA, stratified by race/ethnicity and SEER stage. SEER stage. 0: In situ: noninvasive, intraepithelial; 1: Localized only; 2: Regional by direct extension only; 3: Regional lymph node(s) involved only; 4: Regional by BOTH direct extension AND regional lymph node(s) involved; 5: Regional, NOS; 7: Distant site(s)/lymph node(s) involved; 8: Unknown if extension or metastasis. Models adjusted for pack-years smoked (0, 1-10, 11-20, 21-30, 31-40, >40), alcohol consumption (<1 drinks/month, ≤1 drinks/day, >1 drinks/day), sex (male, female), number of cigarettes smoked per day among smokers (5 cigarettes or less, 6-10 cigarettes, 11-20, 21-30, 31 or more), race/ethnicity (European American, African American, Japanese American, Latin American, Native Hawaiian), and reproductive factors among females. Body mass index (BMI, kg/m3) and smoking status (ever, never, current) were included as strata variables due to their proportional hazard violation. Categorical birthyear was also included as a strata variable to adjust for possible cohort effects. CI: Confidence Interval; HR: Hazard Ratio.

Supplementary Figure S11 Supplementary Figure 11: Associations between Any T2D and BCA by race/ethnicity among never smokers. Models adjusted for alcohol consumption (<1 drinks/month, ≤1 drinks/day, >1 drinks/day), sex (male, female), race/ethnicity (European American, African American, Japanese American, Latin American, Native Hawaiian), and reproductive factors among females. Body mass index (BMI, kg/m3) was included as a strata variable due to a proportional hazard violation. Categorical birthyear was also included as a strata variable to adjust for possible cohort effects. CI: Confidence Interval; HR: Hazard Ratio; P het: P- heterogeneity for likelihood ratio test for difference in association across covariate levels.

Supplementary Table S1 Supplementary Table 1: Global proportional hazards test for exposure and covariates included in the model

Supplementary Table S2 Supplementary Table 2: Poisson model variable coefficients used to estimate absolute risk. Example rate, per 100,000 for 50 year old African Americans with diabetes: 21.6 = exp(-7.23 + 4.31*(log(50/70)) + 0.24*1) * 100,000

Supplementary Table S3 Supplementary Table 3: Race/ethnicity specific incidence rates, per 100,000, adjusted to U.S. standard 2000 population, with left truncation.
