# Supplementary material for: Interethnic Differences in Bladder Cancer Incidence and the Association between Type 2 Diabetes and Bladder Cancer in the Multiethnic Cohort Study
Source: Cancer Res Commun. 2023 May 2;3(5):755–62. doi: 10.1158/2767-9764.CRC-22-0288 (PMC10153456; doi:10.1158/2767-9764.CRC-22-0288)
Supplement: Supplementary Figure S1 — Supplementary Figure 1: MEC participant exclusion, showing removal of participants based on data missingness. [file crc-22-0288-s01.pdf]

Supplementary Figures

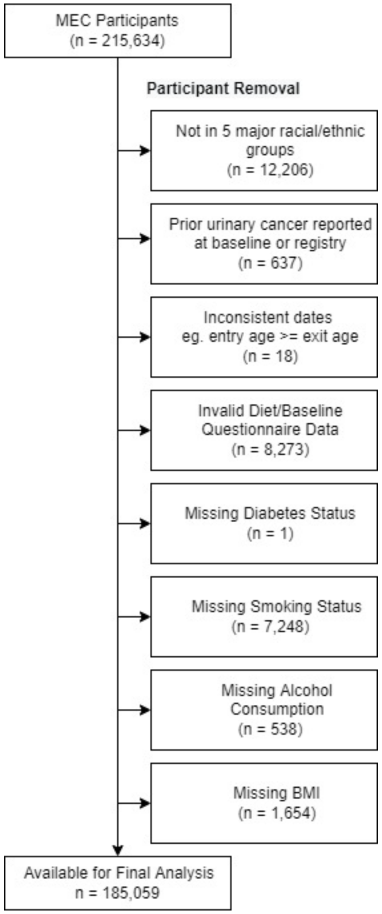

Supplementary Figure 1: MEC participant exclusion, showing removal of participants based on data missingness.
