# Supplementary material for: Interethnic Differences in Bladder Cancer Incidence and the Association between Type 2 Diabetes and Bladder Cancer in the Multiethnic Cohort Study
Source: Cancer Res Commun. 2023 May 2;3(5):755–62. doi: 10.1158/2767-9764.CRC-22-0288 (PMC10153456; doi:10.1158/2767-9764.CRC-22-0288)
Supplement: Supplementary Figure S2 — Supplementary Figure 2: Surveillance, Epidemiology, and End Results Program stage distribution of bladder cancer cases by race/ethnicity. 0: In situ: noninvasive, intraepithelial; 1: Localized only; 2: Regional by direct extension only; 3: Regional lymph node(s) involved only; 4: Regional by BOTH direct extension AND regional lymph node(s) involved; 5: Regional, NOS; 7: Distant site(s)/lymph node(s) involved; 8: Unknown if extension or metastasis; M: Missing. [file crc-22-0288-s02.pdf]

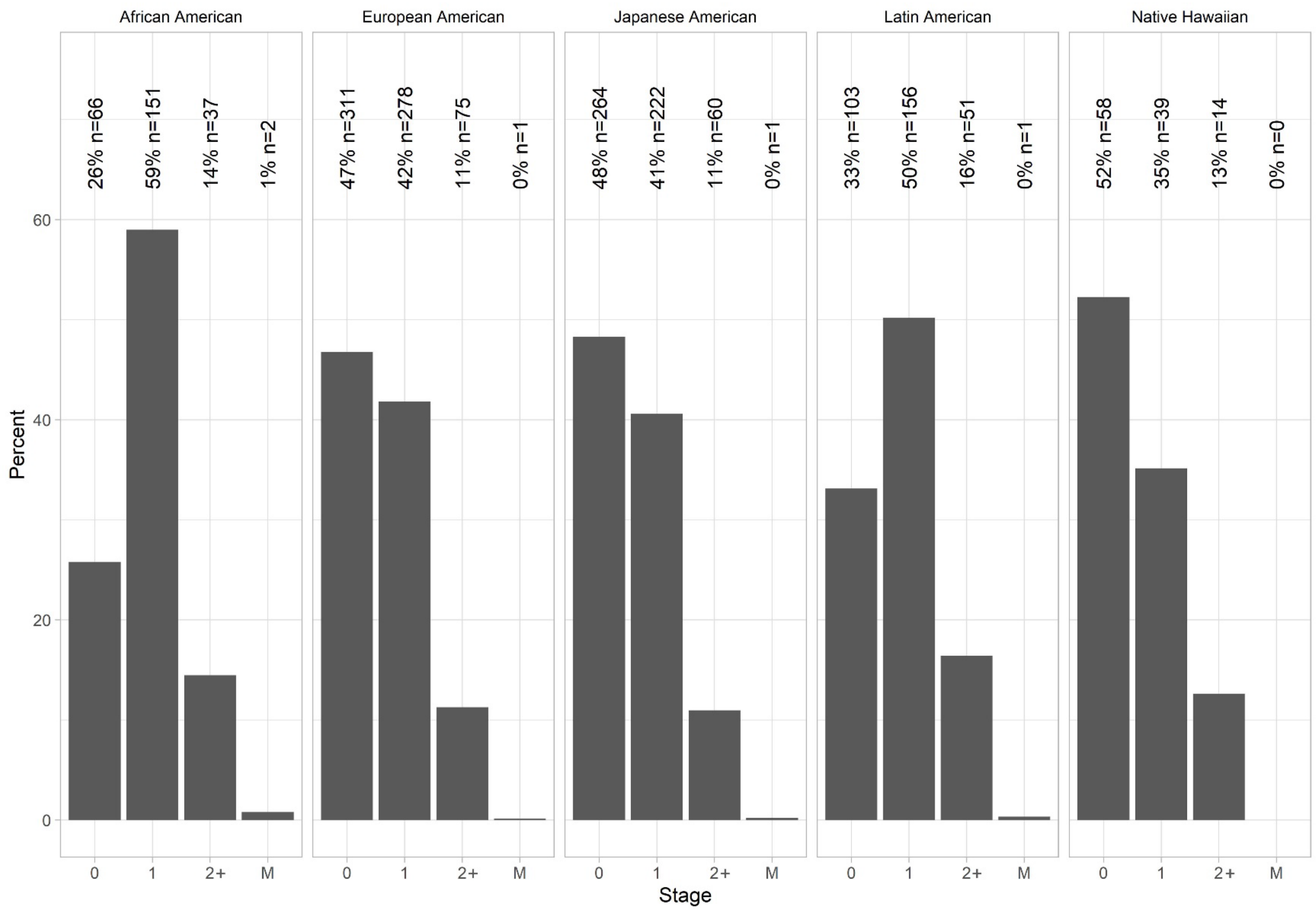

Supplementary Figure 2: Surveillance, Epidemiology, and End Results Program stage distribution of bladder cancer cases by race/ethnicity. 0: In situ: noninvasive, intraepithelial; 1: Localized only; 2: Regional by direct extension only; 3: Regional lymph node(s) involved only; 4: Regional by BOTH direct extension AND regional lymph node(s) involved; 5: Regional, NOS; 7: Distant site(s)/lymph node(s) involved; 8: Unknown if extension or metastasis; M: Missing.
