# Supplementary material for: Interethnic Differences in Bladder Cancer Incidence and the Association between Type 2 Diabetes and Bladder Cancer in the Multiethnic Cohort Study
Source: Cancer Res Commun. 2023 May 2;3(5):755–62. doi: 10.1158/2767-9764.CRC-22-0288 (PMC10153456; doi:10.1158/2767-9764.CRC-22-0288)
Supplement: Supplementary Figure S3 — Supplemental Figure 3: Distribution of MEC participation across participation opportunities by race/ethnicity, represented as percentage by race/ethnicity. BL: baseline questionnaire; FU QX: follow-up questionnaire; BR: biorepository questionnaire; CMS: Medicare FFS. [file crc-22-0288-s03.pdf]

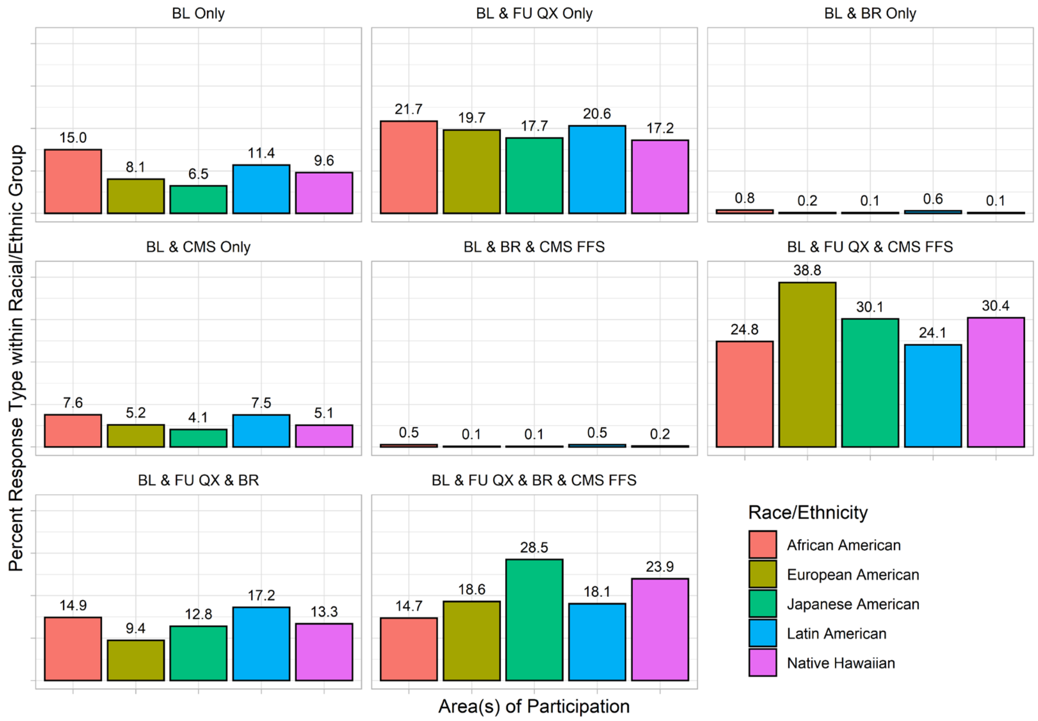

Supplemental Figure 3: Distribution of MEC participation across participation opportunities by race/ethnicity, represented as percentage by race/ethnicity. BL: baseline questionnaire; FU QX: follow-up questionnaire; BR: biorepository questionnaire; CMS: Medicare FFS.
