# Supplementary material for: Interethnic Differences in Bladder Cancer Incidence and the Association between Type 2 Diabetes and Bladder Cancer in the Multiethnic Cohort Study
Source: Cancer Res Commun. 2023 May 2;3(5):755–62. doi: 10.1158/2767-9764.CRC-22-0288 (PMC10153456; doi:10.1158/2767-9764.CRC-22-0288)
Supplement: Supplementary Figure S4 — Supplemental Figure 4: Response rates to follow-up questionnaire among MEC participants who returned the baseline questionnaire and were alive for each respective survey by race/ethnicity. As an example, on the 3rd questionnaire, 44.3% of living African American participants returned a questionnaire. Bar heights shorten based on number of participants alive during the survey collection period. QX: Questionnaire. [file crc-22-0288-s04.pdf]

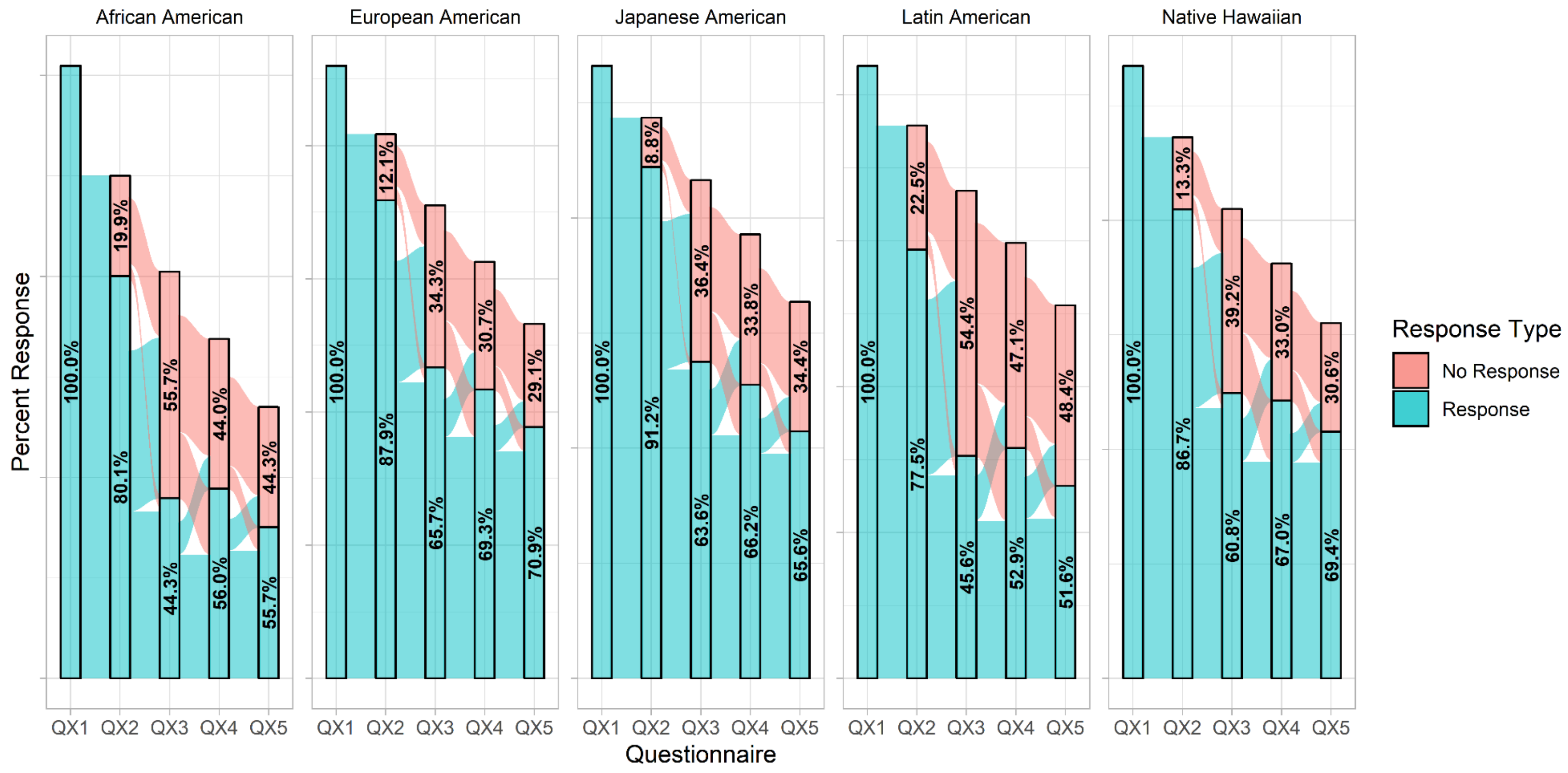

Supplemental Figure 4: Response rates to follow-up questionnaire among MEC participants who returned the baseline questionnaire and were alive for each respective survey by race/ethnicity. As an example, on the 3rd questionnaire, 44.3% of living African American participants returned a questionnaire. Bar heights shorten based on number of participants alive during the survey collection period. QX: Questionnaire.
