# Supplementary material for: Interethnic Differences in Bladder Cancer Incidence and the Association between Type 2 Diabetes and Bladder Cancer in the Multiethnic Cohort Study
Source: Cancer Res Commun. 2023 May 2;3(5):755–62. doi: 10.1158/2767-9764.CRC-22-0288 (PMC10153456; doi:10.1158/2767-9764.CRC-22-0288)
Supplement: Supplementary Figure S11 — Supplementary Figure 11: Associations between Any T2D and BCA by race/ethnicity among never smokers. Models adjusted for alcohol consumption (<1 drinks/month, ≤1 drinks/day, >1 drinks/day), sex (male, female), race/ethnicity (European American, African American, Japanese American, Latin American, Native Hawaiian), and reproductive factors among females. Body mass index (BMI, kg/m3) was included as a strata variable due to a proportional hazard violation. Categorical birthyear was also included as a strata variable to adjust for possible cohort effects. CI: Confidence Interval; HR: Hazard Ratio; P het: P- heterogeneity for likelihood ratio test for difference in association across covariate levels. [file crc-22-0288-s11.pdf]

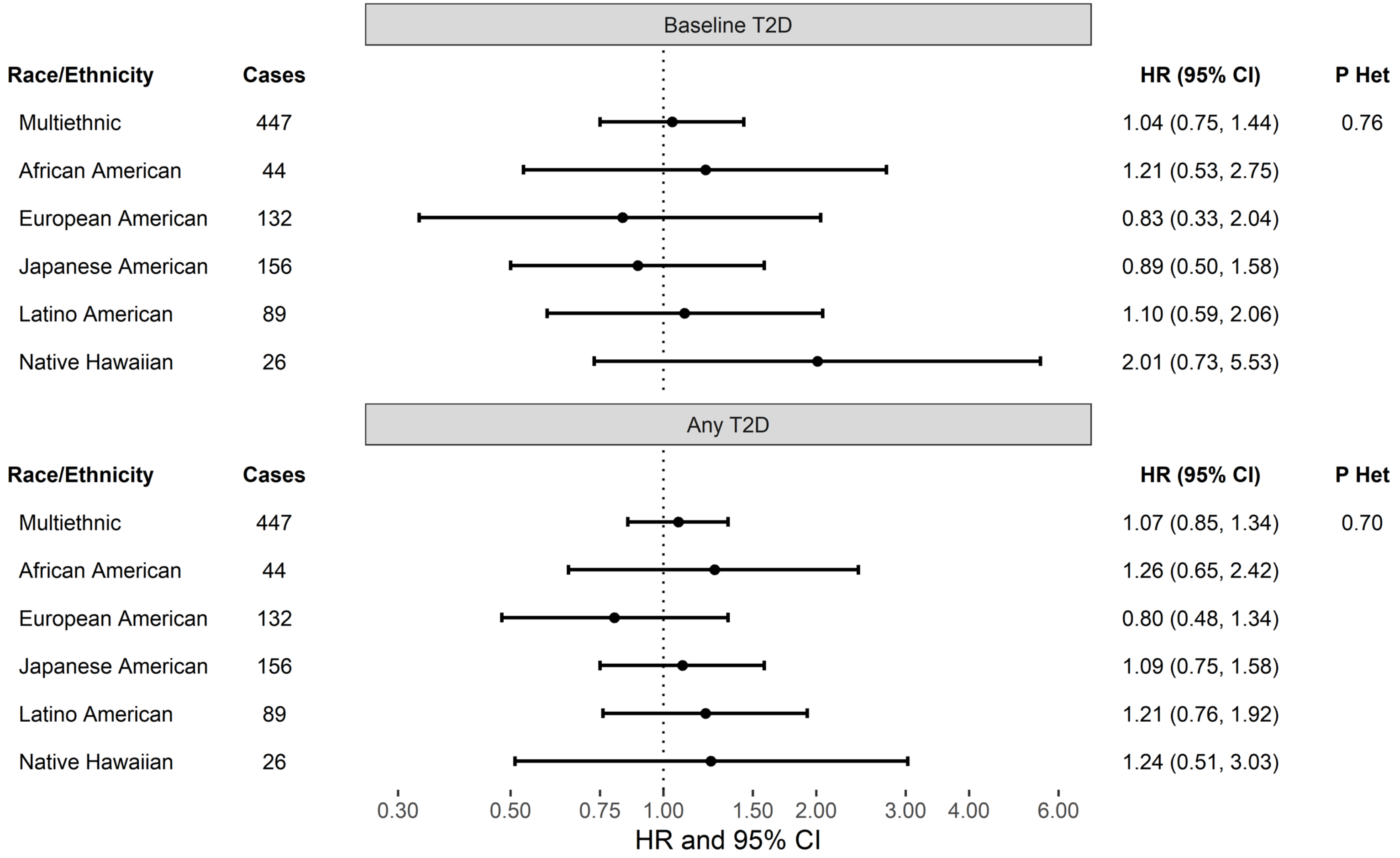

Supplementary Figure 11: Associations between *Any T2D* and BCA by race/ethnicity among never smokers. Models adjusted for alcohol consumption (<1 drinks/month, ≤1 drinks/day, >1 drinks/day), sex (male, female), race/ethnicity (European American, African American, Japanese American, Latin American, Native Hawaiian), and reproductive factors among females. Body mass index (BMI, kg/m^3) was included as a strata variable due to a proportional hazard violation. Categorical birthyear was also included as a strata variable to adjust for possible cohort effects. CI: Confidence Interval; HR: Hazard Ratio; P het: P- heterogeneity for likelihood ratio test for difference in association across covariate levels.
