# Supplementary material for: Interethnic Differences in Bladder Cancer Incidence and the Association between Type 2 Diabetes and Bladder Cancer in the Multiethnic Cohort Study
Source: Cancer Res Commun. 2023 May 2;3(5):755–62. doi: 10.1158/2767-9764.CRC-22-0288 (PMC10153456; doi:10.1158/2767-9764.CRC-22-0288)
Supplement: Supplementary Table S1 — Supplementary Table 1: Global proportional hazards test for exposure and covariates included in the model [file crc-22-0288-s12.pdf]

| **Variable** | **PH Test p[1]** |
|---|---|
| Race/Ethnicity | 3.77E-01 |
| Baseline Diabetes | 1.70E-01 |
| Sex | 5.69E-01 |
| BMI Category | **7.40E-04** |
| Smoking Status | **5.29E-03** |
| Pack-Years | 5.49E-01 |
| Alcohol Consumption | 1.45E-01 |
| Birthyear | 9.34E-01 |
| T2D Any[2] | 5.35E-01 |
| Cigarettes per Day | 7.69E-01 |
| HRT Estrogen Use[3] | 8.29E-01 |
| HRT Progesterone Use[3] | 5.21E-01 |
| Number of Children[3] | 7.08E-01 |
| Menopause Status and Type[3] | 1.52E-01 |

1. Global PH test for all variable levels.
2. Time-varying diabetes measure.
3. PH test among females only.

Supplementary Table 1: Global proportional hazards test for exposure and covariates included in the model.
