# Supplementary material for: Interethnic Differences in Bladder Cancer Incidence and the Association between Type 2 Diabetes and Bladder Cancer in the Multiethnic Cohort Study
Source: Cancer Res Commun. 2023 May 2;3(5):755–62. doi: 10.1158/2767-9764.CRC-22-0288 (PMC10153456; doi:10.1158/2767-9764.CRC-22-0288)
Supplement: Supplementary Table S2 — Supplementary Table 2: Poisson model variable coefficients used to estimate absolute risk. Example rate, per 100,000 for 50 year old African Americans with diabetes: 21.6 = exp(-7.23 + 4.31*(log(50/70)) + 0.24*1) * 100,000 [file crc-22-0288-s13.pdf]

Variable	African American	European American	Japanese American	Latin American	Native Hawaiian
Constant (B0)	-7.80	-7.23	-7.71	-8.05	-7.68
log(age/70)	4.07	4.31	3.64	4.69	4.63
Any T2D	0.18	0.24	0.21	0.29	0.33

Supplementary Table 2: Poisson model variable coefficients used to estimate absolute risk. Example rate, per 100,000 for 50 year old African Americans with diabetes: 21.6 = exp(-7.23 + 4.31*(log(50/70)) + 0.24*1) * 100,000
