# Supplementary material for: Interethnic Differences in Bladder Cancer Incidence and the Association between Type 2 Diabetes and Bladder Cancer in the Multiethnic Cohort Study
Source: Cancer Res Commun. 2023 May 2;3(5):755–62. doi: 10.1158/2767-9764.CRC-22-0288 (PMC10153456; doi:10.1158/2767-9764.CRC-22-0288)
Supplement: Supplementary Table S3 — Supplementary Table 3: Race/ethnicity specific incidence rates, per 100,000, adjusted to U.S. standard 2000 population, with left truncation. [file crc-22-0288-s14.pdf]

All Stage Bladder Cancer Adjusted Incidence Rates

Race/Ethnicity	n	cases	Adjusted Rate per 100,000
Multiethnic	185,059	1,890	35.4
African American	30,481	256	30.5
European American	45,995	665	52.3
Japanese American	53,216	547	33.6
Latin American	41,995	311	24.2
Native Hawaiian	13,372	111	34.5

Supplementary Table 3: Race/ethnicity specific incidence rates, per 100,000, adjusted to U.S. standard 2000 population, with left-truncation.
